# Supplementary material for: Association between dietary inflammatory index and NT-proBNP levels in US adults: A cross-sectional analysis
Source: PLoS One. 2024 Jun 5;19(6):e0304289. doi: 10.1371/journal.pone.0304289 (PMC11152272; doi:10.1371/journal.pone.0304289)
Supplement: S1 Table — (DOCX) [file pone.0304289.s001.docx]

**Table S1.** **Comparison of Each Component of DII Scores Among All Participants.**

| Variables | Overall  (n = 10766) | Non-HF  (n = 10392) | HF  (n = 374) | *P*-value |
| --- | --- | --- | --- | --- |
| Energy | 0.00±0.00 | 0.00±0.00 | -0.07±0.01 | < 0.001 |
| Protein | 0.00±0.00 | 0.00±0.00 | -0.01±0.00 | < 0.001 |
| Carbohydrate | -0.01±0.00 | -0.01±0.00 | -0.05±0.00 | < 0.001 |
| Fiber | 0.24±0.01 | 0.23±0.01 | 0.33±0.02 | < 0.001 |
| Total fatty acid | 0.03±0.00 | 0.03±0.00 | -0.06±0.01 | < 0.001 |
| Total saturated fatty acid | -0.06±0.00 | -0.06±0.00 | -0.17±0.02 | < 0.001 |
| MUFA | 0.00±0.00 | 0.00±0.00 | 0.00±0.00 | < 0.001 |
| PUFA | -0.03±0.00 | -0.03±0.00 | 0.06±0.02 | < 0.001 |
| N-3 Fatty Acids | 0.27±0.00 | 0.27±0.00 | 0.27±0.01 | 0.517 |
| N-6 Fatty Acids | -0.05±0.00 | -0.05±0.00 | -0.02±0.01 | < 0.001 |
| Cholesterol | -0.02±0.00 | -0.02±0.00 | -0.03±0.01 | 0.355 |
| Vitamin A | 0.19±0.00 | 0.19±0.00 | 0.22±0.01 | 0.049 |
| Vitamin B1 | 0.01±0.00 | 0.01±0.00 | 0.03±0.00 | < 0.001 |
| Vitamin B2 | -0.01±0.00 | -0.01±0.00 | 0.00±0.00 | 0.001 |
| Vitamin B6 | -0.06±0.00 | -0.06±0.00 | 0.01±0.02 | < 0.001 |
| Vitamin B12 | -0.02±0.00 | -0.02±0.00 | -0.03±0.00 | 0.002 |
| Vitamin C | 0.17±0.01 | 0.17±0.01 | 0.19±0.02 | 0.418 |
| Vitamin E | 0.19±0.00 | 0.19±0.00 | 0.28±0.01 | < 0.001 |
| Folate | 0.10±0.00 | 0.10±0.00 | 0.13±0.01 | < 0.001 |
| β-Carotene | 0.36±0.01 | 0.36±0.01 | 0.38±0.02 | 0.600 |
| Niacin | 0.04±0.00 | 0.04±0.00 | 0.10±0.01 | < 0.001 |
| Iron | 0.00±0.00 | 0.00±0.00 | -0.01±0.00 | < 0.001 |
| Magnesium | 0.08±0.01 | 0.08±0.01 | 0.17±0.02 | < 0.001 |
| Zinc | -0.02±0.00 | -0.03±0.00 | 0.06±0.02 | < 0.001 |
| Selenium | -0.09±0.00 | -0.09±0.00 | -0.05±0.01 | < 0.001 |
| Caffeine | 0.08±0.00 | 0.08±0.00 | 0.08±0.00 | 0.255 |
| Alcohol | 0.17±0.00 | 0.16±0.00 | 0.22±0.01 | < 0.0001 |

Data are presented the mean and 95% confidence interval. DII, dietary inflammatory index; MUFA, monounsaturated fatty acids; PUFA, polyunsaturated fatty acids.
