# Supplementary material for: Association between dietary inflammatory index and NT-proBNP levels in US adults: A cross-sectional analysis
Source: PLoS One. 2024 Jun 5;19(6):e0304289. doi: 10.1371/journal.pone.0304289 (PMC11152272; doi:10.1371/journal.pone.0304289)
Supplement: S2 Table — (DOCX) [file pone.0304289.s002.docx]

**Table S2.** **Comparison of Each Actual Dietary Component of DII Among All Participants.**

| Variables | Overall  (n = 10766) | Non-HF  (n = 10392) | HF  (n = 374) | *P*-value |
| --- | --- | --- | --- | --- |
| Energy (kcal) | 2223.70±12.63 | 2235.14±12.55 | 1750.01±58.58 | < 0.001 |
| Protein (g) | 82.74±0.61 | 83.11±0.60 | 67.42±2.84 | < 0.001 |
| Carbohydrate (g) | 272.55±1.73 | 273.87±1.72 | 217.90±7.68 | < 0.001 |
| Fiber (g) | 15.75±0.22 | 15.80±0.22 | 13.62±0.36 | < 0.001 |
| Total fatty acid (g) | 83.74±0.49 | 84.15±0.49 | 66.92±2.73 | < 0.001 |
| Total saturated fatty acid (g) | 27.47±0.18 | 27.62±0.18 | 21.25±0.93 | < 0.001 |
| MUFA (g) | 31.42±0.21 | 31.59±0.20 | 24.62±1.13 | < 0.001 |
| PUFA (g) | 17.37±0.14 | 17.44±0.15 | 14.49±0.66 | < 0.001 |
| N-3 Fatty Acids (g) | 0.14±0.01 | 0.14±0.01 | 0.12±0.02 | 0.379 |
| N-6 Fatty Acids (g) | 16.99±0.14 | 17.06±0.14 | 14.21±0.65 | < 0.001 |
| Cholesterol (mg) | 292.21±2.95 | 292.97±2.97 | 260.46±13.61 | 0.022 |
| Vitamin A (mcg) | 617.60±11.48 | 618.79±11.52 | 568.51±34.66 | 0.146 |
| Vitamin B1 (mg) | 1.65±0.02 | 1.65±0.02 | 1.39±0.06 | < 0.001 |
| Vitamin B2 (mg) | 2.15±0.02 | 2.16±0.02 | 1.86±0.08 | < 0.001 |
| Vitamin B6 (mg) | 1.87±0.02 | 1.88±0.02 | 1.56±0.07 | < 0.001 |
| Vitamin B12 (mcg) | 5.22±0.12 | 5.24±0.11 | 4.16±0.35 | 0.001 |
| Vitamin C (mg) | 91.96±2.24 | 92.18±2.28 | 82.85±5.39 | 0.117 |
| Vitamin E (mg) | 7.15±0.08 | 7.19±0.08 | 5.40±0.14 | < 0.001 |
| Folate (mcg) | 192.75±3.46 | 193.93±3.46 | 146.68±9.27 | < 0.001 |
| β-Carotene (mcg) | 1977.44±70.30 | 1983.44±70.29 | 1742.35±171.52 | 0.150 |
| Niacin (mg) | 23.66±0.23 | 23.77±0.23 | 19.07±0.78 | < 0.001 |
| Iron (mg) | 15.63±0.15 | 15.69±0.15 | 13.12±0.49 | < 0.001 |
| Magnesium (mg) | 284.72±2.83 | 285.82±2.85 | 239.32±7.53 | < 0.001 |
| Zinc (mg) | 12.05±0.11 | 12.10±0.11 | 10.20±0.58 | 0.002 |
| Selenium (mcg) | 110.00±0.87 | 110.51±0.87 | 89.00±3.72 | < 0.001 |
| Caffeine (mg) | 201.48±5.26 | 202.06±5.21 | 177.44±19.68 | 0.190 |
| Alcohol (g) | 11.68±0.54 | 11.83±0.56 | 5.48±1.35 | < 0.001 |

Data are presented the mean and 95% confidence interval. DII, dietary inflammatory index; MUFA, monounsaturated fatty acids; PUFA, polyunsaturated fatty acids.
