# Supplementary material for: Association between dietary inflammatory index and NT-proBNP levels in US adults: A cross-sectional analysis
Source: PLoS One. 2024 Jun 5;19(6):e0304289. doi: 10.1371/journal.pone.0304289 (PMC11152272; doi:10.1371/journal.pone.0304289)
Supplement: Attachment — Submitted filename: Answer.docx [file pone.0304289.s003.docx]

Dear Editor and Reviewer(s).

We appreciate the opportunity allowing us to revise our manuscript and thanks for reviewers’ constructive comments and suggestions. We would like to submit our revised manuscript, entitled“**Association between Dietary Inflammatory Index and NT-proBNP Levels in US adults: A Cross-Sectional Analysis**”(Manuscript ID: PONE-D-23-33232) for consideration for publication. In the revised manuscript.we have carefully addressed all comments and questions raised by the reviewer(s) point-by-point. We greatly appreciate your time and efforts to improve our manuscript for publication.

**Reviewer1**

1. Why was NT-proBNP chosen as the molecule to study? How does it compare to other potential biomarkers? Are there better indicators?

Reply1: Dear Reviewer, thank you for your valuable feedback. With the continuous improvement in laboratory testing capabilities, an increasing number of sensitive and specific cardiac biomarkers are being applied clinically. Laboratory tests for cardiac biomarkers are categorized into: markers of myocardial injury, cardiac function markers, inflammatory markers, and markers of myocardial fibrosis, among others. NT-proBNP is a key biomarker of cardiac function, capable of reflecting cardiac stress and dysfunction, and is widely used in clinical practice as a marker of cardiac function. Recent literature has increasingly confirmed that BNP is a "gold standard" for diagnosing chronic heart failure and assessing cardiac function, particularly in differentiating cardiogenic heart failure from pulmonary diseases[1, 2]. Compared to other biomarkers for heart failure, NT-proBNP has high specificity and sensitivity. A negative BNP result can be used to exclude the diagnosis of left ventricular heart failure. Furthermore, the level of NT-proBNP can assess the severity of heart failure and is an independent risk factor for cardiovascular events within one year in severe heart failure cases[3]. Given that this study explores the relationship between dietary inflammation and cardiovascular health, the association of NT-proBNP with inflammatory states makes it an ideal choice for investigating this relationship.

2.Did the authors look at CAD specifically in its analysis?

Reply2: Thank you for your valuable feedback. In our initial manuscript, our analysis was primarily focused on cardiovascular diseases (CVD). However, your query has highlighted the necessity for a more detailed exploration of heart failure (HF). Therefore, in the revised version, we plan to include an additional weighted multivariate regression analysis specifically addressing HF, to provide deeper insights. Moreover, we will add related unweighted analyses in supplementary materials to further enrich our study and have updated and recreated relevant figures to enhance our presentation.

3.In the Discussion on page 9, the authors have mistaken gender to be synonymous with sex. This is incorrect. You did not monitor gender. You only collected data on sex. Please correct this paragraph on page 9.

Reply3: Thank you for pointing out the inaccurate terminology used in the discussion section on page 9. We acknowledge the incorrect use of "sex" and "gender" within our study, as we indeed only collected data on the gender of participants. Therefore, we will correct this section in the revised manuscript to ensure the accuracy and clarity of our terminology.

We marked the changes in red in the revised manuscript.These comments are all valuable and enable us to greatly improve thequality ofour manuscript. We tried our best to improve the manuscript.These changes will not influence the content and framework of thepaper. We appreciate for Editors/Reviewers’warm work earnestly. And hope that the corrections will meet with approval. thank you very much for your comments and suggestions.

4.The authors found no relationship to CVD but continue to discuss it on the first paragraph on page 10. This could be removed as speculation and is not supported statistically by their own data.

Reply4: Thank you for your insightful comments, which have inspired new considerations in our approach. Indeed, our data did not demonstrate a significant relationship with cardiovascular disease (CVD). Therefore, we plan to revise the content on page 10 accordingly. We will include additional analyses on heart failure, especially given that our findings were not pronounced in patients with coronary artery disease but were present in those with heart failure (particularly in unweighted cases). We have reanalyzed our data and recreated the relevant tables and figures. We will also discuss how these results vary across different populations to provide a more comprehensive perspective. Thank you for your valuable input, which has significantly enhanced the quality of our manuscript.

5. I agree whole-heartedly with the limitations identified in lines 312 to 320. They are important, especially the reliability of dietary recalls. Simply identifying the limitations does not remove any of the detrimental significance of these limitations. The authors may want to rebut or at least try to address these limitations in some manner to counter their importance.

Reply5: Thank you for highlighting the limitations identified in our study, particularly concerning the reliability of dietary recall. We acknowledge the impact that the limitations regarding dietary recall reliability may have on our findings. While these limitations cannot be completely overcome in the present study, we have endeavored to mitigate their impact through rigorous statistical methods and multivariate analyses. In future research, we plan to employ more precise methods of dietary data collection, such as prospective dietary logs, to enhance the accuracy and reliability of the data.

**Reviewer2**

1. In “Study Design” section (Line 64): please specify characteristics of the study participants, specifically: minimum and maximum age; number of subjects in the age groups <50 years, 50-75 years, >75 years, divided by gender and ethnicity; inclusion and exclusion criteria based on NT-proBNP and dietary measurements, lifestyle factors, health status and pregnancy status. Reference to Figure 1 is missing in the main text.

Reply6: Dear Reviewer, Thank you very much for your suggestions and guidance. Regarding the detailed explanation of the characteristics of the study participants in the "Study Design" section as you mentioned, we now provide the following additional information in response to your request:

Characteristics of participants: This study integrated data from three NHANES cycles (1999–2004) to evaluate NT-proBNP levels in participants at a mobile examination center, with blood samples preserved for future analysis. After excluding participants for various reasons, including out-of-range NT-proBNP levels (n=365), incomplete dietary data (n=528), self-reported pregnancy (n=627), and absence of HF diagnosis data (n=43), the study population was narrowed down to 10,766 individuals, comprising 5,475 women and 5,313 men. In the women cohort, 2552 participants were under 50, 2117 were aged between 50 and 75, and 784 were over 75. Among the men, 2426 were younger than 50, 2159 were within the 50 and 75 age range, and 728 were older than 75. Additionally, we noted the absence of a reference to Figure 1 in the main text. We will cite Figure 1 in the appropriate section of the revised manuscript to better aid readers in understanding the study design and participant screening process.

2. In “Assessment of NT-proBNP” section (Line 83): measurements of NT-proBNP in serum samples were performed after minimum 14 years, this may raise concerns about storage practices and samples integrity. Please specify samples storage method and conditions. Then, indicate the step-by-step protocol for dosing NT-proBNP.

Reply7:Thank you for your attention to the assessment of NT-proBNP in our study. Following your suggestion, we have revised this section's description and uploaded the relevant original experimental documents as supplementary files[4]. We have revised the description to read: Serum samples from the 1999-2004 NHANES cycles were analyzed for NT-proBNP levels, with the assays conducted between 2018 and 2020 at the University of Maryland School of Medicine’s laboratory, under the leadership of Dr. Roche Diagnostics. The NHANES Biospecimen Program provided protocols for sample handling, detailing the processes for processing and storage. NT-proBNP measurements were performed using the Roche Cobas e601 analyzer (Elecsys, Roche Diagnostics), adhering to established detection limits (5 pg/mL to 35,000 pg/mL) and exhibiting a coefficient of variation of 3.1% at a low concentration (46 pg/mL) and 2.7% at a high concentration (32,805 pg/mL). A consistent detection threshold was employed to ensure the accuracy and reliability of the measurements. Additionally, prior research has demonstrated the stability of NT-proBNP in samples stored for extended periods[5-7].Thank you for your suggestion, which has now made this section of the manuscript clearer.

3. In “Assessment of Dietary Inflammatory Index” section (Line 91): starting from the 24-hour dietary recall survey, describe in details data gathering, encoding and refinement process. Please, list all the 27 dietary factors used in the DII and describe how it was possible to calculate their intakes (frequencies of consumption, portions, reference nutritional databases). Describe how the DII is structured and the criteria for which the DII score was obtained. Describe the clinical significance of the division into quartiles of DII scores, and“Assessment of Food Groups and Nutrients Intake” section: it is important to present information on individual food groups, foods and nutrients intake of the study participants, to evaluate dietary intake contributions on relationships between DII and NT-proBNP levels. Please, add related analyses in the “Stratified Analysis” section (Line 201).

Reply8: Thank you for your attention in our study. In this study, participants' dietary intake data were collected via a 24-hour dietary recall method. Through the Mobile Examination Center (MEC), detailed records of the food and beverages consumed by participants in the 24 hours preceding the interview were documented. The reported dietary components were subsequently quantified using the USDA Food and Nutrient Database for Dietary Studies to determine the content of specific nutrients.

DII Components

this research was based on 27 dietary components known to modulate inflammation, including energy, protein, carbohydrates, fiber, total fats, saturated fats, monounsaturated and polyunsaturated fatty acids, cholesterol, a range of vitamins and minerals including vitamin A, β-carotene, thiamin, riboflavin, niacin, vitamin B6, folic acid, vitamin B12, vitamin C, vitamin E, magnesium, iron, zinc, and selenium, caffeine, alcohol, and n-3 and n-6 fatty acids

DII Calculation Method

Each participant's dietary intake was matched to a comprehensive database encompassing global average daily intake figures and their standard deviations for each dietary component across 45 populations worldwide. The intake of each nutrient was contrasted with its global average and subsequently divided by the standard deviation to calculate the Z-scores for each nutrient. These Z-scores were converted into percentiles, centered around zero by doubling and then subtracting one, yielding scores from -1 to +1. Each centered score was multiplied by its respective dietary component’s inflammation effect score to calculate specific DII scores for each food parameter. The overall DII score for an individual was calculated by aggregating these specific DII scores.

Clinical Significance

The division into quartiles of DII scores was utilized to reflect levels of dietary inflammation. Higher DII scores typically indicate a pro-inflammatory diet, while lower DII scores suggest an anti-inflammatory diet. Previous research, as well as our study, has demonstrated significant associations between the DII and inflammatory markers such as Klotho and C-reactive protein (CRP), confirming the clinical relevance of DII scoring[8].

Additionally, following the review of your comments, we recognized the importance of providing more information on food categories and nutrient intakes. We plan to include a lasso regression of the original data constituting the DII in the revised manuscript, and Figure 4 will display the association of each component with NT-proBNP. We will also further elucidate in the text why we believe that focusing on the overall DII score is appropriate. While decomposing the DII components for individual analysis may have its value in certain contexts, in the backdrop of our study, we believe that concentrating on the overall DII score better captures the holistic impact of dietary patterns on inflammation and heart health. This aligns with the original intent of our study design, which is to assess the impact of overall dietary patterns rather than single foods or nutrients on heart health.

We believe such additions will offer a more comprehensive view to our readers, aiding in their understanding. In summary, we look forward to addressing your comments more comprehensively through these revisions, thereby enhancing the depth and breadth of our study.

4.In “Baseline Characteristics of Patients” section (Line 158): p-values in Table 1 refer to inter-quartile differences, therefore the statements from line 164 to 167 require a post-hoc analysis to be statistically supported. From line 162 to 164 ("In patients with...pg/mL.") the sentences refer to Table 2 and not to Table 1. In line 168 the p-value related to C-reactive protein corresponds to <0.001 and not to <0.01 (as shown in Table 2). Tables 1 and 2 present chaotic content and poor captions. Please implement them to ensure a better understanding of the tables.

Reply9: Thank you for your thorough review and valuable suggestions. Regarding the issues raised with Tables 1 and 2, we have made the following amendments and restructuring to the description of the results section:

Baseline Characteristics of Participants:

Table 1 presents the baseline characteristics of the study population, divided into quartiles (Q1-Q4) based on their Dietary Inflammatory Index (DII) scores to examine variable distribution. Out of 10,766 participants, 374 reported a history of heart failure (HF). Those with HF had a mean age of approximately 64.77 ± 1.16 years, with a gender distribution of 48.26% women and 51.74% men. Racial and ethnic backgrounds predominantly included non-Hispanic whites (76.87%), with the remainder being Mexican Americans (3.23%), non-Hispanic blacks (10.76%), and other races (9.14%). Chronic diseases were prevalent in the HF group, with chronic kidney disease (CKD) present in 51.01%, hypertension in 70.48%, hyperlipidemia in 86.71%, and diabetes in 34.18%.

For the 9,392 participants without a history of HF, stratification by DII scores revealed distinct quartiles, showing an increasing percentage of women and a decreasing percentage of men with higher DII quartiles. The racial and ethnic distribution among the non-HF participants closely mirrored that of the HF cohort. The prevalence of chronic diseases such as CKD, hypertension, and hyperlipidemia increased with higher DII quartiles. Weighted linear regression analyses identified significant associations between DII and demographic variables, including age, gender, and race or ethnicity (gender p < 0.001, race or ethnicity p < 0.001), showing distinct variations across DII quartiles. A significant increase in body mass index (BMI) was observed with higher DII quartiles (p < 0.001), though waist circumference remained relatively constant across the groups (p = 0.370).

Table 2 highlights that, in patients with HF, the mean level of N-terminal pro b-type natriuretic peptide (NT-proBNP) was significantly elevated, recorded at 1035.53 ± 113.63 pg/mL. Other cardiovascular risk factors measured included albumin, high-density lipoprotein cholesterol (HDL-C), low-density lipoprotein cholesterol (LDL-C), triglycerides, and total cholesterol, with mean values of 4.16 ± 0.02 g/dL, 1.24 ± 0.03 mg/dL, 2.93 ± 0.08 mg/dL, 171.50 ± 5.62 mg/dL, and 197.14 ± 2.85 mg/dL, respectively. Furthermore, mean levels of glycated hemoglobin and C-reactive protein (CRP) were reported as 5.99 ± 0.07% and 0.77 ± 0.06 mg/dL, respectively.

In individuals without a history of HF, stratification by DII quartiles revealed significant inter-group differences in the mean levels of NT-proBNP, albumin, HDL-C, LDL-C, triglycerides, and total cholesterol. NT-proBNP and CRP levels showed substantial correlations with DII quartiles (NT-proBNP P-value = 0.002, CRP P-value < 0.001), highlighting a considerable association with other cardiac metabolic biomarkers.

Corrections have been made to accurately reference Table 2 instead of Table 1 for the content mentioned from lines 162 to 164.

The p-value related to C-reactive protein mentioned in line 168 has been corrected according to the data presented in Table 2 for accuracy. With these modifications, we believe the content of Tables 1 and 2 is now clearer and more comprehensible, with descriptions that are more fitting. This will aid readers in better understanding the study results.

5.In “Relationships of Dietary Inflammatory Index (DII) and NT-proBNP” section (Line 173): in lines 175 and 177 the reported p-value is wrong (see Table 3). Concerning the sentence in lines 191-192, see the comment related to lines 164-167.

Reply10: We have thoroughly revised the table, and the corrected version is now presented as Table 3: Associations between Dietary Inflammatory Index (DII) and NT-proBNP levels.

The spline smoothing curves in Fig 2 visually depict a positive correlation between the DII and NT-proBNP levels. Our regression analyses, as summarized in the revised Table 3, aimed to assess the association between DII and NT-proBNP concentrations. For participants without a history of heart failure (HF), an incremental unit increase in DII was associated with a significant elevation in NT-proBNP levels across different models: an increase of 9.69 pg/dL in the crude model (95% confidence interval [CI]: 6.47, 12.91; p < 0.001), 8.57 pg/dL in Model I (95% CI: 4.97, 12.17; p < 0.001), and 5.44 pg/dL in Model II (95% CI: 1.51, 9.38; p = 0.011) with further adjustments. The quartile-based analysis examined the associations between DII and NT-proBNP levels across the quartiles, indicating an upward trend in NT-proBNP levels with increasing DII. Specifically in Model II, NT-proBNP levels in Q4 were increased by 16.76 pg/dL (95% CI: -6.73, 40.25; p = 0.153), compared to Q1, suggesting a trend where higher DII corresponds to elevated NT-proBNP levels, albeit with reduced significance upon adjustment. For individuals with a history of HF, Model II analysis showed a notable and statistically insignificant correlation between DII levels in Q4 and increased NT-proBNP levels, with a β-value of 162.69 pg/dL (95% CI: -14.64, 340.03; p = 0.071).

Moreover, the unweighted analysis in Table S1 emphasizes the positive correlation between DII and NT-proBNP levels in individuals with a history of HF. In Model II, the increase in NT-proBNP levels in Q4 was significant, with a β-value of 925.9 pg/dL (95% CI: 118.58, 1733.21; p = 0.032).

We have ensured that the reported p-values align correctly with the data presented in the revised Table 3. Additionally, we have addressed the related comments regarding the sentences .We appreciate your diligence in reviewing our work and apologize for any confusion caused by the oversight.

6.In “Discussion” chapter (Line 217): please, enrich discussion contents by adding a greater number of comparisons between results obtained in your study and those from other studies. Moreover, deepen the discussion regarding negative results obtained. Deepen the influence of dietary factors constituting the DII on cholesterol, triglycerides and glycohemoglobin serum parameters.

Reply10: Thank you very much for your thorough review and valuable suggestions. We have revised the discussion section in accordance with your guidance, aiming to provide a more comprehensive comparison of our study results with those of other research and a more in-depth exploration of negative findings. Additionally, we have further discussed the impact of dietary factors constituting the DII on related diseases.

The revised text is as follows: Previous studies have highlighted the association between DII scores and CVD morbidity, emphasizing the significant impact of dietary inflammation on health[9]. Our analysis identifies proteins, carbohydrates, dietary fibers, monounsaturated fatty acids, polyunsaturated fatty acids, vitamins A, β-carotene, thiamine, niacin, folate, vitamin E, magnesium, iron, selenium, caffeine, alcohol, and n-3 fatty acids as vital dietary factors impacting NT-proBNP levels. For instance, protein and amino acid supplementation may improve muscle mass and functional capacity in patients with HF. However, evidence is still emerging on its effects across different types of HF, such as HF with preserved ejection fraction (HFpEF)[10, 11]. Low-carbohydrate diets have demonstrated positive outcomes for patients with diabetic cardiomyopathy, improving symptoms and overall quality of life associated with HF. In a randomized controlled trial, patients with diabetic cardiomyopathy who followed a low-carbohydrate diet exhibited significant weight and systolic blood pressure reductions over 16 weeks[12]. Moreover, studies indicate the beneficial role of polyunsaturated fatty acids, particularly n-3 fatty acids, in cardiovascular health. Their anti-inflammatory and antioxidant properties are credited with lowering the risk of CVD and HF[13, 14]. Dietary fiber, particularly from plant sources, plays a crucial role in cardiac metabolic stability, contributing to a reduced risk of HF. This benefit is assumed to stem from fiber’s ability to modulate inflammatory biomarkers[15].

The role of micronutrients such as iron, magnesium, and selenium in HF prognosis is critical. Iron deficiency, prevalent among patients with HF, has been linked to increased cardiovascular hospitalization rates, particularly in those with HF with reduced fraction(HFrEF)[16, 17]. Magnesium’s role is pivotal in developing HF [18], with deficiencies exacerbating the condition. Selenium’s antioxidant properties are vital for tissue protection against oxidative stress[19]. Vitamins A and β-carotene, thiamine (B1), niacin (B3), folate, and vitamin E are crucial for HF management and overall cardiovascular health, though their specific effects warrant further investigation. The association between alcohol intake and HF risk is nuanced, with some studies suggesting potential cardiovascular benefits from moderate alcohol consumption. though evidence remains inconclusive regarding a direct causal relationship[20, 21].

These revisions aim to address your valuable feedback and enhance the depth and breadth of the discussion. We appreciate your guidance and the opportunity to refine our manuscript.

7.In “Abstract” (Line 11): in line 17, the statement "multivariable adjusted analyses" is partly correct. Specify that "non-adjusted analysis" was also performed.

Reply11: Thank you for your careful review and valuable feedback. We have revised the Abstract to address your concern and explicitly mention the inclusion of non-adjusted analyses. The revised version is as follows:

Background: With cardiovascular diseases standing as a leading cause of mortality worldwide, the interplay between diet-induced inflammation, as quantified by the Dietary Inflammatory Index (DII), and heart failure biomarker NT-proBNP has not been investigated in the general population.

Methods: This study analyzed data from the National Health and Nutrition Examination Survey (NHANES) 1999-2002, encompassing 10,766 individuals. The relationship between the DII and NT-proBNP levels was evaluated through multivariable-adjusted regression models. To pinpoint crucial dietary components influencing NT-proBNP levels, the LASSO regression model was utilized. Stratified analyses were then conducted to examine the associations within specific subgroups to identify differential effects of the DII on NT-proBNP levels across diverse populations.

Results: In individuals without heart failure, a unit increase in the DII was significantly associated with an increase in NT-proBNP levels. Specifically, NT-proBNP levels rose by 9.69 pg/dL (95% CI: 6.47, 12.91; p < 0.001) without adjustments, 8.57 pg/dL (95% CI: 4.97, 12.17; p < 0.001) after adjusting for demographic factors, and 5.44 pg/dL (95% CI: 1.51, 9.38; p = 0.011) with further adjustments for health and lifestyle variables. In participants with a history of heart failure, those in the highest DII quartile showed a trend towards higher NT-proBNP levels compared to those in the lowest quartile, although this difference did not reach statistical significance (β = 162.69 pg/dL; 95% CI: -14.64, 340.03; p = 0.071). Significant interactions were observed in subgroup analyses by age, gender, hypertension, and diabetes.

Conclusion: This study identified a positive correlation between the DII and NT-proBNP levels, suggesting a robust link between pro-inflammatory diets and increased heart failure biomarkers, with implications for dietary modifications in cardiovascular risk management.

8. In “Introduction” chapter (Line 33): from lines 34 to 37 the content is extremely generic and does not provide useful information for understanding the study. From lines 39 to 43 concepts are expressed in a redundant way. Please, add other useful indexes to evaluate associations between the effects of dietary patterns and cardiovascular health. In Lines 50-51, please specify what is meant by "changes in ventricular wall stress". From line 57 to 60 references are missing.

Reply11: Thank you for your valuable suggestions. We have revisited the "Introduction" chapter, incorporating your feedback to enhance clarity and relevance. Additionally, we have included references where needed. The revised version is presented below:

Over recent decades, cardiovascular diseases (CVD) have risen to become a leading global health concern. The World Health Organization identifies CVD as the primary cause of mortality worldwide, responsible for millions of fatalities each year[22, 23]. The surge in heart failure (HF) cases, propelled by an aging demographic, highlights the critical need for extensive research into cardiovascular health. However, HF frequently develops insidiously, with significant symptoms emerging only upon the occurrence of severe health episodes. Over time, the heart experiences alterations due to myocardial cell remodeling. In older individuals, this remodeling can result in a progressive deterioration of heart function, ultimately leading to HF[24]. Therefore, identifying risk factors for HF is essential for its prevention, early diagnosis, and management.

Chronic inflammation is recognized as a pivotal risk factor in the intricate causality of CVDs[25, 26]. Recent investigations into the Dietary Inflammatory Index (DII) have revealed a significant association between the DII and cardiovascular health. The DII evaluates the inflammatory impact of dietary components, offering insights into chronic inflammation and the risk of CVD[27]. Since its inception in 2009 and subsequent update in 2014, the DII has individually tested the diet-induced inflammatory potential[28, 29]. The DII’s objective is to provide a comprehensive analysis of the inflammatory effects of various dietary components, thereby determining the overall inflammatory potential of the diet.

Conventionally, the diagnosis of HF has relied on symptoms and sign evaluation. However, the last two decades have seen N-terminal pro B-type natriuretic peptide (NT-proBNP) measurement emerge as a superior diagnostic criterion for HF[2, 3]. NT-proBNP, a sensitive indicator of cardiac strain and dysfunction, is closely associated with the incidence and severity of HF. Its role extends to monitoring therapeutic outcomes and predicting the disease prognosis[30-33]. offering a dynamic and precise measure of cardiac health status beyond conventional diagnostic practices.

This study aimed to investigate the association between the DII and NT-proBNP levels utilizing data from the National Health and Nutrition Examination Survey (NHANES 1999-2002).

9.In “Methods” and “Results” chapters (Line 63/156): choose the term "gender" instead of "sex" in the main text and tables; choose between the terms "unadjusted model" or "crude model" and use the one chosen all the time. Review the table titles. -In “Discussion” chapter (Line 217): choose the terms "men" and "women" instead of "males" and "females". From line 271 to 273 and from line 278 to 280 references are missing.

Reply12: Thank you for bringing the terminology issue to our attention. We appreciate your guidance. In the revised manuscript, we have made the necessary corrections, consistently using "gender" instead of "sex" throughout the main text and tables. Additionally, we have opted for the term "crude model" uniformly in the manuscript. We believe these adjustments will enhance the accuracy and clarity of our presentation. Please feel free to let us know if there are any further considerations or if additional changes are required.

We marked the changes in red in the revised manuscript.These comments are all valuable and enable us to greatly improve thequality ofour manuscript. We tried our best to improve the manuscript.These changes will not influence the content and framework of thepaper. We appreciate for Editors/Reviewers’warm work earnestly. And hope that the corrections will meet with approval. Please do not hesitate to contact us with any further questions or recommendations. Once again. thank you very much for your comments and suggestions.

**Reference**

1.Jensen J, Ma LP, Fu ML, Svaninger D, Lundberg PA, Hammarsten O. Inflammation increases NT-proBNP and the NT-proBNP/BNP ratio. Clin Res Cardiol. 2010;99(7):445-52. Epub 2010/03/17. doi: 10.1007/s00392-010-0140-z. PubMed PMID: 20229122.

2.McKie PM, Burnett JC, Jr. NT-proBNP: The Gold Standard Biomarker in Heart Failure. J Am Coll Cardiol. 2016;68(22):2437-9. Epub 2016/12/03. doi: 10.1016/j.jacc.2016.10.001. PubMed PMID: 27908348.

3.Schmitt W, Rühs H, Burghaus R, Diedrich C, Duwal S, Eissing T, et al. NT-proBNP Qualifies as a Surrogate for Clinical End Points in Heart Failure. Clin Pharmacol Ther. 2021;110(2):498-507. Epub 2021/02/26. doi: 10.1002/cpt.2222. PubMed PMID: 33630302; PubMed Central PMCID: PMCPMC8360001 development of heart failure treatment.

4.Centers for Disease Control. National Health and Nutrition Examination Survey. Data Codebook. 1999-2004. <https://wwwn.cdc.gov/Nchs/Nhanes/1999-2000/SSBNP_A.htm#Analytic_Notes>. Accessed 1 September 2023.

5.van der Merwe DE, Henley R, Lane G, Field R, Frenneaux M, Dunstan F, et al. Effect of different sample types and stability after blood collection of N-terminal pro-B-type natriuretic peptide as measured with Roche Elecsys system. Clin Chem. 2004;50(4):779-80. Epub 2004/03/27. doi: 10.1373/clinchem.2003.025064. PubMed PMID: 15044344.

6.Cauliez B, Guignery J, Marinier S, Mariau I, Lavoinne A. Two-year stability of NT-proBNP in frozen samples using the Roche Elecsys system. Ann Clin Biochem. 2008;45(Pt 3):318-9. Epub 2008/05/17. doi: 10.1258/acb.2007.007187. PubMed PMID: 18482923.

7.Parrinello CM, Grams ME, Couper D, Ballantyne CM, Hoogeveen RC, Eckfeldt JH, et al. Recalibration of blood analytes over 25 years in the atherosclerosis risk in communities study: impact of recalibration on chronic kidney disease prevalence and incidence. Clin Chem. 2015;61(7):938-47. Epub 2015/05/09. doi: 10.1373/clinchem.2015.238873. PubMed PMID: 25952043; PubMed Central PMCID: PMCPMC4782184.

8.Ma TC, Zhou J, Wang CX, Fang M, Gao F. Association Between Dietary Inflammatory Index and S-Klotho Plasma Levels in Middle-Aged and Elderly People. Front Nutr. 2022;9:853332. Epub 2022/05/28. doi: 10.3389/fnut.2022.853332. PubMed PMID: 35619959; PubMed Central PMCID: PMCPMC9127966.

9.Ho FK, Wirth MD, Parra-Soto S, Boonpor J, Zhou Z, Petermann-Rocha F, et al. Dose-Response Associations of Dietary Inflammatory Potential With Health Outcomes: A Prospective Cohort Study of 198,265 UK Biobank Participants. Curr Probl Cardiol. 2023;48(9):101774. Epub 2023/05/01. doi: 10.1016/j.cpcardiol.2023.101774. PubMed PMID: 37121456.

10.Rozentryt P, von Haehling S, Lainscak M, Nowak JU, Kalantar-Zadeh K, Polonski L, et al. The effects of a high-caloric protein-rich oral nutritional supplement in patients with chronic heart failure and cachexia on quality of life, body composition, and inflammation markers: a randomized, double-blind pilot study. J Cachexia Sarcopenia Muscle. 2010;1(1):35-42. Epub 2011/04/09. doi: 10.1007/s13539-010-0008-0. PubMed PMID: 21475692; PubMed Central PMCID: PMCPMC3060643.

11.Deutz NE, Matheson EM, Matarese LE, Luo M, Baggs GE, Nelson JL, et al. Readmission and mortality in malnourished, older, hospitalized adults treated with a specialized oral nutritional supplement: A randomized clinical trial. Clin Nutr. 2016;35(1):18-26. Epub 2016/01/23. doi: 10.1016/j.clnu.2015.12.010. PubMed PMID: 26797412.

12.Kleissl-Muir S, Owen A, Rasmussen B, Zinn C, Driscoll A. Effects of a low carbohydrate diet on heart failure symptoms and quality of life in patients with diabetic cardiomyopathy: A randomised controlled trial pilot study. Nutr Metab Cardiovasc Dis. 2023;33(12):2455-63. Epub 2023/10/06. doi: 10.1016/j.numecd.2023.08.015. PubMed PMID: 37798235.

13.Djoussé L, Cook NR, Kim E, Bodar V, Walter J, Bubes V, et al. Supplementation With Vitamin D and Omega-3 Fatty Acids and Incidence of Heart Failure Hospitalization: VITAL-Heart Failure. Circulation. 2020;141(9):784-6. Epub 2019/11/12. doi: 10.1161/circulationaha.119.044645. PubMed PMID: 31709816; PubMed Central PMCID: PMCPMC7054158.

14.Tavazzi L, Maggioni AP, Marchioli R, Barlera S, Franzosi MG, Latini R, et al. Effect of n-3 polyunsaturated fatty acids in patients with chronic heart failure (the GISSI-HF trial): a randomised, double-blind, placebo-controlled trial. Lancet. 2008;372(9645):1223-30. Epub 2008/09/02. doi: 10.1016/s0140-6736(08)61239-8. PubMed PMID: 18757090.

15.Marques FZ, Nelson E, Chu PY, Horlock D, Fiedler A, Ziemann M, et al. High-Fiber Diet and Acetate Supplementation Change the Gut Microbiota and Prevent the Development of Hypertension and Heart Failure in Hypertensive Mice. Circulation. 2017;135(10):964-77. Epub 2016/12/09. doi: 10.1161/circulationaha.116.024545. PubMed PMID: 27927713.

16.Graham FJ, Pellicori P, Kalra PR, Ford I, Bruzzese D, Cleland JGF. Intravenous iron in patients with heart failure and iron deficiency: an updated meta-analysis. Eur J Heart Fail. 2023;25(4):528-37. Epub 2023/02/25. doi: 10.1002/ejhf.2810. PubMed PMID: 36823953.

17.Docherty KF, Welsh P, Verma S, De Boer RA, O'Meara E, Bengtsson O, et al. Iron Deficiency in Heart Failure and Effect of Dapagliflozin: Findings From DAPA-HF. Circulation. 2022;146(13):980-94. Epub 2022/08/17. doi: 10.1161/circulationaha.122.060511. PubMed PMID: 35971840; PubMed Central PMCID: PMCPMC9508991.

18.Voultsos P, Bazmpani MA, Papanastasiou CA, Papadopoulos CE, Efthimiadis G, Karvounis H, et al. Magnesium Disorders and Prognosis in Heart Failure: A Systematic Review. Cardiol Rev. 2022;30(6):281-5. Epub 2021/05/19. doi: 10.1097/crd.0000000000000397. PubMed PMID: 34001688.

19.Bomer N, Grote Beverborg N, Hoes MF, Streng KW, Vermeer M, Dokter MM, et al. Selenium and outcome in heart failure. Eur J Heart Fail. 2020;22(8):1415-23. Epub 2019/12/07. doi: 10.1002/ejhf.1644. PubMed PMID: 31808274; PubMed Central PMCID: PMCPMC7540257.

20.Soukoulis V, Dihu JB, Sole M, Anker SD, Cleland J, Fonarow GC, et al. Micronutrient deficiencies an unmet need in heart failure. J Am Coll Cardiol. 2009;54(18):1660-73. Epub 2009/10/24. doi: 10.1016/j.jacc.2009.08.012. PubMed PMID: 19850206.

21.McKeag NA, McKinley MC, Harbinson MT, Noad RL, Dixon LH, McGinty A, et al. The effect of multiple micronutrient supplementation on left ventricular ejection fraction in patients with chronic stable heart failure: a randomized, placebo-controlled trial. JACC Heart Fail. 2014;2(3):308-17. Epub 2014/06/24. doi: 10.1016/j.jchf.2013.12.008. PubMed PMID: 24952700.

22.Roth GA, Mensah GA, Johnson CO, Addolorato G, Ammirati E, Baddour LM, et al. Global Burden of Cardiovascular Diseases and Risk Factors, 1990-2019: Update From the GBD 2019 Study. J Am Coll Cardiol. 2020;76(25):2982-3021. Epub 2020/12/15. doi: 10.1016/j.jacc.2020.11.010. PubMed PMID: 33309175; PubMed Central PMCID: PMCPMC7755038.

23.Mensah GA, Roth GA, Fuster V. The Global Burden of Cardiovascular Diseases and Risk Factors: 2020 and Beyond. J Am Coll Cardiol. 2019;74(20):2529-32. Epub 2019/11/16. doi: 10.1016/j.jacc.2019.10.009. PubMed PMID: 31727292.

24.McDonagh TA, Metra M, Adamo M, Gardner RS, Baumbach A, Böhm M, et al. 2021 ESC Guidelines for the diagnosis and treatment of acute and chronic heart failure: Developed by the Task Force for the diagnosis and treatment of acute and chronic heart failure of the European Society of Cardiology (ESC). With the special contribution of the Heart Failure Association (HFA) of the ESC. Eur J Heart Fail. 2022;24(1):4-131. Epub 2022/01/28. doi: 10.1002/ejhf.2333. PubMed PMID: 35083827.

25.Adamo L, Rocha-Resende C, Prabhu SD, Mann DL. Reappraising the role of inflammation in heart failure. Nat Rev Cardiol. 2020;17(5):269-85. Epub 2020/01/24. doi: 10.1038/s41569-019-0315-x. PubMed PMID: 31969688.

26.Silvestre-Roig C, Braster Q, Ortega-Gomez A, Soehnlein O. Neutrophils as regulators of cardiovascular inflammation. Nat Rev Cardiol. 2020;17(6):327-40. Epub 2020/01/31. doi: 10.1038/s41569-019-0326-7. PubMed PMID: 31996800.

27.Hariharan R, Odjidja EN, Scott D, Shivappa N, Hébert JR, Hodge A, et al. The dietary inflammatory index, obesity, type 2 diabetes, and cardiovascular risk factors and diseases. Obes Rev. 2022;23(1):e13349. Epub 2021/10/29. doi: 10.1111/obr.13349. PubMed PMID: 34708499.

28.Cavicchia PP, Steck SE, Hurley TG, Hussey JR, Ma Y, Ockene IS, et al. A new dietary inflammatory index predicts interval changes in serum high-sensitivity C-reactive protein. J Nutr. 2009;139(12):2365-72. Epub 2009/10/30. doi: 10.3945/jn.109.114025. PubMed PMID: 19864399; PubMed Central PMCID: PMCPMC2777480.

29.Shivappa N, Steck SE, Hurley TG, Hussey JR, Hébert JR. Designing and developing a literature-derived, population-based dietary inflammatory index. Public Health Nutr. 2014;17(8):1689-96. Epub 2013/08/15. doi: 10.1017/s1368980013002115. PubMed PMID: 23941862; PubMed Central PMCID: PMCPMC3925198.

30.Januzzi JL, van Kimmenade R, Lainchbury J, Bayes-Genis A, Ordonez-Llanos J, Santalo-Bel M, et al. NT-proBNP testing for diagnosis and short-term prognosis in acute destabilized heart failure: an international pooled analysis of 1256 patients: the International Collaborative of NT-proBNP Study. European heart journal. 2006;27(3):330-7.

31.de Bold AJ, Bruneau BG, Kuroski de Bold ML. Mechanical and neuroendocrine regulation of the endocrine heart. Cardiovasc Res. 1996;31(1):7-18. Epub 1996/01/01. PubMed PMID: 8849584.

32.Echouffo-Tcheugui JB, Zhang S, Daya N, McEvoy JW, Tang O, Juraschek SP, et al. NT-proBNP and All-Cause and Cardiovascular Mortality in US Adults: A Prospective Cohort Study. J Am Heart Assoc. 2023;12(11):e029110. Epub 2023/05/26. doi: 10.1161/jaha.122.029110. PubMed PMID: 37232235; PubMed Central PMCID: PMCPMC10382006.

33.Hamatani Y, Nagai T, Shiraishi Y, Kohsaka S, Nakai M, Nishimura K, et al. Long-Term Prognostic Significance of Plasma B-Type Natriuretic Peptide Level in Patients With Acute Heart Failure With Reduced, Mid-Range, and Preserved Ejection Fractions. Am J Cardiol. 2018;121(6):731-8. Epub 2018/02/06. doi: 10.1016/j.amjcard.2017.12.012. PubMed PMID: 29394996.
