# Supplementary material for: Association between dietary inflammatory index and NT-proBNP levels in US adults: A cross-sectional analysis
Source: PLoS One. 2024 Jun 5;19(6):e0304289. doi: 10.1371/journal.pone.0304289 (PMC11152272; doi:10.1371/journal.pone.0304289)
Supplement: Attachment — Submitted filename: Answer2.docx [file pone.0304289.s004.docx]

Dear Editor and Reviewer.

We are grateful for the opportunity to revise our manuscript entitled "Association between Dietary Inflammatory Index and NT-proBNP Levels in US adults: A Cross-Sectional Analysis" (Manuscript ID: PONE-D-23-33232) and would like to resubmit it for consideration. We have carefully addressed all comments and questions raised by the reviewer(s) in a point-by-point manner. Thank you for your time and efforts in helping us improve our manuscript for publication.

1.Please explain how you were able to detect in non-HF patients (Table 1) levels of NT-proBNP

from 95.06±7 pg/dL (so 0,95 pg/mL) to 129.91±7.04 pg/dL (so 1.2 pg/mL) if you report that minimum detection level is set at 5 pg/mL. Please, check unit of measure for NT-proBNP in HF subjects. Please also be aware that the unit of measure you report for HDL and LDL levels in Table 1 is wrong; not being aware of their meaning would be a serious concern for publication.

We sincerely appreciate your thorough review and the critical issues you have brought to our attention. Your comments have been invaluable in identifying areas that require clarification and correction, ultimately enhancing the quality of our manuscript.

Upon careful re-examination of the data, we acknowledge the discrepancies in the NT-proBNP levels reported in Table 1. We will rectify this error by ensuring all values are consistently expressed in pg/mL, in line with the assay's minimal detection level of 5 pg/mL. Furthermore, we will meticulously review the entire manuscript to guarantee that all NT-proBNP data is accurately presented and discussed.

Regarding the incorrect units for HDL and LDL levels, we deeply regret this oversight and understand the potential implications it may have on the interpretation of our findings. We will promptly amend the table to reflect the correct units and will diligently verify that all biomarker data throughout the manuscript is accurately represented.

We are committed to maintaining the highest standards of scientific integrity and accuracy in our research. Your feedback has been a crucial reminder of the importance of rigorous data presentation and interpretation. Rest assured that we will implement more stringent review processes to prevent such errors from occurring in our future work.

2.Student's t test and chi-square test for comparing less than 3 groups at a time. I therefore believe that for the analyzes reported in Table 1 and Table 2 for non-HF subjects, one-way ANOVA (normally distributed data) or Kruskal-Wallis (non-normally distributed data) would be appropriate. Then, please use these suggested tests in your analyses.Please indicate in all tables the specific statistical test used, and results as mean and SD were appropriate.

Thank you for your meticulous review and valuable suggestions. In response to your comments regarding the appropriateness of statistical methods, we have reconsidered our analysis strategy. Initially, Table 1 did indeed involve four DII groups, for which one-way ANOVA or the Kruskal-Wallis test is generally recommended to assess intergroup differences. However, following your guidance and considering the specific distribution of our data, we have now revised our grouping method, simplifying it into two main categories: those with a history of heart failure and those without (Non-HF). With this new grouping strategy, for the comparisons between these two groups, we employed Student's t-test (for normally distributed continuous data) and the chi-square test (for categorical data). These methods are more suitable for comparing mean differences and proportions between two independent samples. All statistical results have been updated to ensure the correct application of statistical tests, and each test's p-value has been clearly indicated in the tables. The results are now reported as mean ± standard deviation for normally distributed data and as actual numbers and weighted percentages for categorical variables.

We appreciate your guidance in refining our statistical approach and data presentation. Your suggestions have undoubtedly strengthened the manuscript, and we are grateful for your thorough review.

3.- Please, in your adjusted analyses use only those variables underlying significant interactions (e.g, age, gender, CKD, hypertension, diabetes).

Thank you very much for your detailed review and suggestions regarding our analysis method. We agree with your point that only those variables that show significant interaction (such as age, gender, CKD, hypertension, and diabetes) should be used in the adjustment of the analysis.

Based on your suggestion, we plan to redo our analysis. In the revised Model II, we will limit the adjusting variables to age, gender, ethnicity, CKD, hypertension, and diabetes. This step will help us understand more accurately the relationship between these variables and the study outcomes and strengthen our conclusions.

The relevant tables will be updated to reflect these adjustments, and more precise data interpretations will be provided in the paper. We believe these changes will enhance the quality and credibility of our study.

4.Please, in partial correlation analyses use Spearman’s coefficient for non-normally distributed data or Pearson’s coefficient for normally distributed data.

Thank you for your thoughtful suggestion regarding the use of Spearman's or Pearson's correlation coefficients in our analyses, depending on the normality of the data distribution. We appreciate your attention to this important methodological consideration.

However, we would like to respectfully point out that the complex nature of the NHANES dataset, which involves intricate sampling designs and weighting procedures, poses limitations to the straightforward application of simple correlation analyses. These methods often do not account for the sample weights, which are crucial in ensuring the representativeness of the findings. Previous NHANES literature rarely uses Spearman's coefficient for non-normally distributed data or Pearson's coefficient for normally distributed data[1-4].

Moreover, in our study, we have prioritized the use of multivariate regression models to control for multiple confounding factors. Given the presence of numerous potential influencing variables, relying solely on correlation analyses (such as Pearson's or Spearman's) may not suffice to uncover the true relationships between the variables of interest. Multivariate analyses, on the other hand, provide a more comprehensive perspective by simultaneously considering the effects of multiple factors.

As demonstrated in "Table 2. Relationship between Dietary Inflammatory Index and NT-proBNP," we have conducted thorough multivariate modeling to examine the associations between the DII and NT-proBNP levels, while adjusting for relevant covariates such as age, gender, race/ethnicity, hypertension, diabetes, and chronic kidney disease. This approach allows for a more robust assessment of the relationships, taking into account the complex interplay of various factors.

We hope that this explanation clarifies our rationale for the analytical methods employed in our study. Rest assured that we have carefully considered the appropriateness of the statistical techniques used, given the specific characteristics of the NHANES dataset and the research questions at hand.

Thank you once again for your valuable input and for engaging in this scientific discourse.

5.Abstract: “Stratified analyses were then conducted to examine the associations within specific subgroups to identify differential effects of the DII on NT-proBNP levels across diverse populations.”; “In individuals without heart failure, a unit increase in the DII was significantly associated with an increase in NT-proBNP levels.”; “Significant interactions were observed in subgroup analyses by age, gender, hypertension, and diabetes.” Please, for a quick understanding by riders, explicitly indicate what these specific subgroups and diverse populations, and a unit increase in the DII, and subgroups analyses are.

Thank you for your insightful comments and suggestions regarding our manuscript. We appreciate the opportunity to clarify the details of our analyses and findings for a more comprehensive understanding. We have revised our abstract to explicitly detail the specific subgroups and diverse populations investigated, as well as to clarify the implications of a unit increase in the DII and our approach to subgroup analyses. The amendments are as follows:

Abstract

Background: With cardiovascular diseases standing as a leading cause of mortality worldwide, the interplay between diet-induced inflammation, as quantified by the Dietary Inflammatory Index (DII), and heart failure biomarker NT-proBNP has not been investigated in the general population.

Methods: This study analyzed data from the National Health and Nutrition Examination Survey (NHANES) 1999-2004, encompassing 10,766 individuals. The relationship between the DII and NT-proBNP levels was evaluated through multivariable-adjusted regression models. To pinpoint crucial dietary components influencing NT-proBNP levels, the LASSO regression model was utilized. Stratified analyses were then conducted to examine the associations within specific subgroups to identify differential effects of the DII on NT-proBNP levels across diverse populations.

Results: In individuals without heart failure, a unit increase in the DII was significantly associated with an increase in NT-proBNP levels. Specifically, NT-proBNP levels rose by 9.69 pg/mL (95% CI: 6.47, 12.91; p < 0.001) without adjustments, 8.57 pg/mL (95% CI: 4.97, 12.17; p < 0.001) after adjusting for demographic factors, and 5.54 pg/mL (95% CI: 1.75, 9.32; p = 0.001) with further adjustments for health variables. In participants with a history of heart failure, those in the second and third DII quartile showed a trend towards higher NT-proBNP levels compared to those in the lowest quartile, with increases of 717.06 pg/mL (95% CI: 76.49-1357.63, p = 0.030) and 855.49 pg/mL (95% CI: 156.57-1554.41, p = 0.018). Significant interactions were observed in subgroup analyses by age (<50: β=3.63, p=0.141; 50-75: β=18.4, p<0.001; >75: β=56.09, p<0.001), gender (men: β=17.82, p<0.001; women: β=7.43, p=0.061),hypertension (β=25.73, p<0.001) and diabetes (β=38.94, p<0.001).

Conclusion: This study identified a positive correlation between the DII and NT-proBNP levels, suggesting a robust link between pro-inflammatory diets and increased heart failure biomarkers, with implications for dietary modifications in cardiovascular risk management.

Thank you for your careful review of our manuscript and for bringing this discrepancy to our attention. We sincerely apologize for the oversight in our reporting regarding the NHANES data cycles.

6. It is reported in the abstract and in line 62 that your analyses refer to data from the NHANES and collected in 1999-2002; however, in lines 77 and 88 you indicate data from three NHANES cycles 1999-2004; so, please indicate the start/end date (year) of each cycle.

Thank you for your careful review of our manuscript and for bringing this discrepancy to our attention. We sincerely apologize for the oversight in our reporting regarding the NHANES data cycles.

We have rectified this error in the revised manuscript. To clarify, the NHANES data utilized in our analyses were collected across three cycles: 1999-2000, 2001-2002, and 2003-2004. We have updated the relevant sections of the manuscript to accurately reflect the start and end dates of each cycle.

We appreciate your diligence in ensuring the accuracy and integrity of our research findings. Please let us know if you require any further clarification or if there are any additional concerns.

7. Please add data indicating the quantification (mean and SD) of each dietary component of the DII, and DII scores (continuous data) and DII quartiles, for HF and non-HF subjects.Please add data indicating the quantification (mean and SD) of each dietary component of the DII, and DII scores (continuous data) and DII quartiles, for HF and non-HF subjects.Please indicate maximum and minimum values of the DII score; and indicate the inflammation effect for each dietary component.

Thank you for your detailed review and for the suggestion to provide a more detailed presentation of the data. In response to your request, we have added comprehensive data detailing the quantification of each dietary component within the Dietary Inflammatory Index as well as the DII scores (continuous data) and DII quartiles, for both HF and non-HF subjects.

During our analysis, we found that the DII scores ranged from a minimum of -4.28 to a maximum of 5.18. Furthermore, the inflammatory effect of each dietary component has also been presented in these tables to offer an intuitive understanding of the impact of each component on the overall DII score. We have meticulously presented this data in tables included in the appendix:

Table S1: Provides the relationship between various dietary components and heart failure status (HF and non-HF), including means and standard deviations.

Table S2: Provides the corresponding data for the DII scores of the actual dietary components related to DII scores with heart failure status.

We believe these additional data and tables fully address your query and enhance the transparency and credibility of our research.

8.In line 290: use “in preserving cardiovascular health” instead of management; then, specify from where is obtained the value of 14.32 pg/dL.

Thank you for your valuable suggestions. I have made the change on line 290, replacing "management" with "in preserving cardiovascular health" as you directed. Regarding the source of the value of 14.32 pg/dL, this figure is derived from the pooled population analysis presented in the forest plot of our manuscript. It based on a consolidated analysis of the data from all study participants included. This approach ensures a comprehensive and representative result, providing an accurate estimate that reflects the overall trend.

9.In lines 77-78: “After excluding participants for various reasons”; please, be specific and don’t use any form such as “various reasons”.

Thank you for pointing out the need for specificity in describing the exclusion criteria for our study participants. In accordance with your suggestion, I have revised lines 77-78 of the manuscript to precisely state the reasons for participant exclusion. The revised text is as follows:

"Participants were excluded based on the following criteria: out-of-range NT-proBNP levels (n=365), incomplete dietary data (n=528), self-reported pregnancy (n=627), and absence of heart failure diagnosis data (n=43). Following these exclusions, the study population was narrowed down to 10,766 individuals."With gratitude for your thorough review and guidance.

10.Please change the section title “Assessment of covariates” in “Assessment of study participants’ characteristics”; please delete the first sentence in lines 119-120.

Thank you for your guidance on refining the section titles and content within our manuscript. As per your suggestions, the section title previously named “Assessment of covariates” has been changed to “Assessment of study participants’ characteristics” to more accurately reflect the content of the section.

Additionally, the first sentence in lines 119-120 has been removed in accordance with your instructions. We believe these changes improve the clarity and focus of the manuscript, and we are grateful for your constructive feedback.

11. Please, explain the reasons of using DII quartiles instead of DII continuous data in your analyses. Please, indicate that by using DII continuous data it did not produce significant results, or add these results. Please, indicate that using DII quartiles reduced inter-individual variability leading to significant findings.

Thank you for your interest in the methodological choices of our study. In this research, we opted to use quartiles of the Dietary Inflammatory Index (DII) rather than solely relying on continuous data for analysis. This approach is principally because quartiles can more effectively reveal the relationships between variables, especially when potential nonlinear relationships are not apparent in the analysis of continuous data, a method that has been widely utilized in past research on NHANES. By categorizing individuals into different DII quartiles, variations in regression coefficients across levels highlighted the differential impact of explanatory variables on response variables. This grouping reduced inter-individual variability and unveiled significant associations that were not detected in the continuous analysis.

Our analysis demonstrates that utilizing DII quartiles offers deeper insights into how dietary inflammation impacts cardiovascular health across different levels. This method allows us to more comprehensively understand the influence of explanatory variables on the trends of response variables, thereby enhancing the interpretability of our research findings.

12. In line 143-144-145: please, don’t use the term segmented. Also, the cut off for DII quartiles you report are wrong (Q1: DII ≤ 144 0.753; Q2: 0.753 < DII ≤ 2.172; Q3: 2.172 < DII ≤ 3.193; Q4: DII 3.193). Please don’t use the term variances.

Thank you for your valuable comments on the choice of methodology and data presentation in our study. We have made the following revisions in response to your suggestions:

We have replaced the term "segmented" with "categorized" in the original text to more accurately describe the process of grouping DII scores.

Regarding the quartile cutoffs you mentioned, we acknowledge that the previous quartiles were based on the DII from the complete NHANES dietary data for the general population, which might have caused slight discrepancies. Therefore, we took your advice into consideration and decided to recalibrate the DII quartile divisions using the dietary data of our study's sample population. The new quartile definitions are as follows:

Q1(Lowest): DII ≤ 0.321

Q2: 0.321 < DII ≤ 1.752

Q3: 1.752 < DII ≤ 2.816

Q4(Highest): DII > 2.816

We have re-conducted the statistical analysis based on these new divisions and updated the corresponding tables accordingly.

We have replaced the term "variances" with "differences" in the original text when exploring the baseline characteristics between DII scores and NT-proBNP levels, to more accurately convey our analytical intent.

13. In line 156: BMI categories cut-off are usually different from those you report, please specify the reason why of these cut-offs you chose.

Thank you for pointing out the issue regarding the BMI categorization cut-offs in our manuscript. You correctly noted that the BMI categories we reported deviate from the conventional standards. Following your suggestion, we revisited the method of categorizing BMI and decided to adopt the more universally accepted standard of using BMI 25 as the cut-off.

This adjustment aligns our study with internationally recognized standards, facilitating a better understanding and comparison of our results by readers. In light of this new categorization, we have re-analyzed our data and updated the relevant tables to reflect this change.

We believe these modifications will enhance the transparency and comparability of our research and ensure that our findings are consistent with widely accepted weight status classification standards.

14. Please add in Table 1 result from comparison between DII scores and NT-proBNP levels (continuous data) also for non-HF subjects.

Thank you very much for your meticulous review and valuable suggestions on our research. In response to your advice, we have reconsidered how to more clearly present the comparison between DII scores and NT-proBNP levels (continuous data), especially among participants without a history of heart failure .

After a thorough analysis, we believe that adjusting the original quartile grouping method to a grouping based on the presence or absence of HF not only makes the data presentation more intuitive but also more clearly reveals the association between heart failure status, DII scores, and NT-proBNP levels. Therefore, we have made corresponding modifications to Table 1, which now shows the baseline characteristics of all participants, Non-HF participants, and HF participants. This change enables readers to more easily understand the health status of participants under different heart failure conditions and their relationship with the dietary inflammatory index.

Regarding the relationship between DII scores and NT-proBNP levels, we have conducted a more exhaustive investigation through multiple regression models in subsequent analyses (as shown in Table 2). These models cover both heart failure and non-heart failure participants and consider other potential confounders, providing a deeper understanding of the relationship between DII scores and NT-proBNP levels.

We believe that these modifications not only better address your suggestions but also provide readers with a clearer and more comprehensive perspective to understand the interactions and associations between heart failure status, dietary inflammatory index, and NT-proBNP levels.

Thank you again for your valuable suggestions and support for our research.

15. In lines 180-181: “stratification by DII scores revealed distinct quartiles.” Please, be explain the term distinct.

Thank you for your inquiry regarding the clarification of the term "distinct" in lines 180-181 of our manuscript, where we mentioned "stratification by DII scores revealed distinct quartiles." We appreciate the opportunity to address this point and refine our presentation of the data.

Upon reflection, we acknowledge the ambiguity that the term "distinct" may have introduced in describing our quartile-based stratification. Our intention was to convey that the quartiles formed by stratification according to DII scores were clearly separable, each representing a unique subset of the study population with different levels of dietary inflammation. However, we recognize that our initial choice of wording might not have adequately communicated the specificity of these groups.

To address this and in response to the feedback received, we have revised our approach to the presentation of our data. Instead of relying on quartiles, we have opted for a more straightforward comparison by reanalyzing the data according to the presence or absence of heart failure status. This has led to the development of Table 1, which now serves as the foundation for a clearer and more detailed description of our study population.

In the revised section, we present the baseline characteristics of the study population:Table 1 presents the baseline characteristics of the study population, stratified by HF status. Of 10,766 participants, 374 reported a history of HF. The mean age of those with HF was approximately 64.77 ± 1.16 years, with a gender distribution of 48.26% women and 51.74% men. Racial and ethnic backgrounds were predominantly non-Hispanic whites (76.87%), with the remainder being Mexican Americans (3.23%), non-Hispanic blacks (10.76%), and other races (9.14%). The prevalence of CKD, hypertension, hyperlipidemia, and diabetes was significantly higher in the HF group compared to the non-HF group (all p < 0.001). Furthermore, smoking status and drinking habits also differed significantly between the two groups (both p < 0.001), with a higher proportion of former smokers and former drinkers in the HF group.

BMI and waist circumference were significantly higher in the HF group compared to the non-HF group (both p < 0.001). Additionally, the mean DII score was significantly higher in the HF group (1.87 ± 0.09) compared to the non-HF group (1.39 ± 0.04) (p < 0.001).NT-proBNP levels were markedly elevated in the HF group (1035.53 ± 112.38 pg/mL) compared to the non-HF group (115.94 ± 4.88 pg/mL, p < 0.001). Moreover, the HF group had significantly lower serum albumin and HDL-C levels, and higher glycohemoglobin, and C-reactive protein levels (all p < 0.001). LDL-C levels were lower in the HF group (p = 0.012), but total cholesterol levels did not differ significantly between the two groups (p = 0.092).

By adopting this revised approach, we aim to provide a clearer and more accurate representation of our study population, facilitating a better understanding of the relationships between dietary inflammation, as measured by DII scores, and heart failure. We believe this adjustment not only addresses the concern raised but also enriches the manuscript by offering a more nuanced analysis of the data.

We hope this clarification meets the reviewer's request and enhances the overall clarity and quality of our manuscript. Thank you for allowing us the opportunity to improve our work.

16. In line 189: “NT-proBNP level was significantly elevated”; please indicate that are elevated in comparison to NT-proBNP in non-HF subjects, and provide references for this statement.

Thank you for your constructive feedback regarding the presentation of NT-proBNP levels in our manuscript. We acknowledge the oversight in our initial submission and have since taken measures to address this by refining our analysis and presentation of data related to NT-proBNP levels. Specifically, we have abandoned the previous quartile-based approach in favor of a more straightforward comparison between subjects with and without heart failure (HF).

To clarify, we have updated Table 1 to accurately reflect the NT-proBNP levels, which were significantly higher in the HF group, with values recorded at 1035.53 ± 112.38 pg/mL, in stark contrast to the non-HF group, which presented levels at 115.94 ± 4.88 pg/mL (p < 0.001). This comparison unequivocally highlights the elevated NT-proBNP levels in HF subjects, providing a clearer understanding of the biomarker's role in this context.

Further, we have expanded our manuscript to include a detailed description of the study population's baseline characteristics, stratified by HF status, as outlined in your request. This encompasses demographic information, prevalence of comorbid conditions, lifestyle factors, and crucial biochemical parameters, all of which underscore the significant differences between the HF and non-HF groups across multiple facets.

These enhancements to our manuscript not only address your concerns but also enrich the narrative by offering a comprehensive overview of the study population, thereby reinforcing the significance of our findings related to NT-proBNP levels in HF patients. We believe these revisions will greatly improve the manuscript's clarity and value to the readership. Thank you once again for your insightful comments, which have been instrumental in refining our work.

17. In lines 210-212: your statement sounds speculative, only results from model I indicate significance.

Thank you for your insightful comment regarding the statement in lines 210-212 of our manuscript. We appreciate your feedback, as it has allowed us to re-evaluate our analysis and strengthen the presentation of our findings.

In response to your suggestion, we have carefully revisited our statistical models and adjusted for potential confounding factors, including age, gender, race/ethnicity, hypertension, diabetes, and chronic kidney disease. After conducting a thorough re-analysis and updating our tables and results section, we can provide a more accurate representation of our findings.

Specifically, Our regression analyses, presented in Table 2, assessed the association between DII and NT-proBNP concentrations. For participants without a history of HF, an incremental unit in DII was associated with a significant elevation in NT-proBNP levels across different models: an increase of 9.69 pg/mL in the crude model (95% confidence interval [CI]: 6.47, 12.91; p < 0.001), 8.57 pg/mL in Model I (95% CI: 4.97, 12.17; p < 0.001), and 5.44 pg/mL in Model II (95% CI: 1.51, 9.38; p = 0.011) with further adjustments. When DII scores were categorized into quartiles, individuals in the highest quartile (DII≥2.816) had significantly higher NT-proBNP levels compared to those in the lowest quartile (DII<0.321) 43.99pg/mL in the crude model (95%CI: 24.26-63.73, p<0.001), 38.22pg/mL in Model Ⅰ (95%CI: 16.05-60.38, p=0.001), and 25.18 pg/mL in Model Ⅱ (95%CI: 3.04-47.32, p=0.027).

In individuals with HF, no significant association was found between DII scores and NT-proBNP levels in the crude model (β=73.46, 95%CI: -72.99-219.90, p=0.317), Model Ⅰ (β=52.8, 95%CI: -79.85-185.46, p=0.424), or Model Ⅱ (β=106.16, 95%CI: -52.50-264.82, p=0.182). However, notable differences in NT-proBNP levels were observed among participants in the second and third quartiles compared to those in the lowest quartile in Model II, with increases of 717.06 pg/mL (95% CI: 76.49-1357.63, p = 0.030) and 855.49 pg/mL (95% CI: 156.57-1554.41, p = 0.018), respectively.

By incorporating the adjustments you recommended, we believe our results have become more robust and accurately reflect the associations observed in our study population. We appreciate your valuable feedback, which has undoubtedly improved the quality and clarity of our manuscript.

18. In line 265: “incorporating 27 dietary factors and three covariates”; please, indicate why you use those covariates, maybe you would meant you used covariates of model I.

Thank you for your inquiry regarding the selection of covariates specified in line 265 of our manuscript. In response to your question, the choice to incorporate age, gender, and race/ethnicity as covariates in our analysis was informed by established precedents in the field, as evidenced by previous research[3, 5]. These studies suggest that adjusting for age, gender, and race/ethnicity in lasso regression equations can significantly enhance the model's accuracy and reliability by accounting for potential confounding factors. Our decision to use these covariates was therefore driven by a desire to adhere to best practices in statistical modeling within the context of our research domain. We believe that this approach not only strengthens the validity of our findings but also ensures consistency with established methodologies in the field.

19. In line 272: please change your sentence in “Out of n=27 dietary variables constituting the DII scores, n=17 show an interaction to NT-proBNP levels in whole population, such as: protein, etc.”.

Thank you for your suggestion. We have revised the sentence in line 272 as follows:

"Out of n=27 dietary variables constituting the DII scores, n=17 show an interaction to NT-proBNP levels in whole population, such as: Protein, carbohydrates, fiber, monounsaturated and polyunsaturated fatty acids, vitamin A, β-carotene, thiamin, niacin, folic acid, vitamin E, magnesium, iron, selenium, caffeine, alcohol, and n-3 fatty acids , which were identified as the dietary factors substantially correlated with NT-proBNP levels (log-transformed, pg/dL)."

We hope this revision adequately addresses your concern and provides a clearer description of the specific dietary factors found to interact with NT-proBNP levels in our study population. Please let us know if you have any further questions or suggestions.

20. In line 49; 90-91; 111; 116 reference is missing.

Thank you for your valuable feedback on our manuscript. We have carefully reviewed your comments and made the necessary adjustments to address the missing references.

Regarding the reference in line 49, we have added two relevant citations to support the association between the Dietary Inflammatory Index (DII) and cardiovascular health. These include a cross-sectional study by Zhou et al[3]. (2022) and a study by Mao et al[5]. (2024) using NHANES data from 1999-2018.

For lines 90-91, we have included a reference to the National Health and Nutrition Examination Survey Biospecimen Program[6], which provides detailed protocols for sample handling, processing, and storage.

Lastly, for lines 111 and 116, we have added two key references related to the development and validation of the DII. The first is a study by Shivappa et al. (2014) that thoroughly validates the DII[7], and the second is another study by Shivappa et al. (2014) demonstrating the predictive accuracy of the DII using a maximum of 30 food parameters[8].

We appreciate your attention to detail and believe that these additional references will strengthen the manuscript. Please let us know if you have any further questions or concerns.

21.In line 52: “The DII objective” is correct and “The DII’s objective” is not; in line 142: “the study complex” is correct and “the study’s complex” is not; in line 188: “Table 2 highlights” is correct and “Table 2 highlighted” is not.

Thank you for your thorough review and valuable feedback on the manuscript. I have carefully considered your suggestions and made the necessary corrections accordingly. The possessive form has been removed from "The DII objective" and "the study complex" to ensure grammatical accuracy. Additionally, the verb tense in the phrase referring to Table 2 has been changed for consistency and clarity.I appreciate your attention to detail and the time you have invested in reviewing this work.

22.In line 90: who is Dr. Roche Diagnostics?

Thank you for raising this question regarding the mention of Dr. Roche Diagnostics in line 90. Upon careful review of the official NHANES documentation, it has come to our attention that this particular stored specimen study was conducted under the supervision of Principal Investigator Dr. Elizabeth Selvin from the Johns Hopkins Bloomberg School of Public Health. Furthermore, the measurements were carried out between 2018 and 2020 at the University of Maryland School of Medicine in Baltimore, Maryland, under the direction of Lab Director Dr. Robert Christenson. Considering the lack of relevance of the name "Dr. Roche Diagnostics" to the content of the article, we have made the decision to remove this reference in the revised manuscript. We appreciate your attention to detail and the opportunity to clarify this information.

We would like to express our great appreciation to you and reviewers for comments on our paper. Looking forward to hearing from you.

Thank you and best regards.

Yours sincerely,

Tengchi Ma

***Reference***

1.Yu Y, Cheng S, Huang H, Deng Y, Cai C, Gu M, et al. Joint association of sedentary behavior and vitamin D status with mortality among cancer survivors. BMC Med. 2023;21(1):411. Epub 2023/10/31. doi: 10.1186/s12916-023-03118-9. PubMed PMID: 37904126; PubMed Central PMCID: PMCPMC10617233.

2.Xiao Q, Cai B, Yin A, Huo H, Lan K, Zhou G, et al. L-shaped association of serum 25-hydroxyvitamin D concentrations with cardiovascular and all-cause mortality in individuals with osteoarthritis: results from the NHANES database prospective cohort study. BMC Med. 2022;20(1):308. Epub 2022/09/21. doi: 10.1186/s12916-022-02510-1. PubMed PMID: 36127705; PubMed Central PMCID: PMCPMC9490951.

3.Zhou N, Xie ZP, Liu Q, Xu Y, Dai SC, Lu J, et al. The dietary inflammatory index and its association with the prevalence of hypertension: A cross-sectional study. Front Immunol. 2022;13:1097228. Epub 2023/02/07. doi: 10.3389/fimmu.2022.1097228. PubMed PMID: 36741368; PubMed Central PMCID: PMCPMC9893776.

4.Iranpour S, Sabour S. Inverse association between caffeine intake and depressive symptoms in US adults: data from National Health and Nutrition Examination Survey (NHANES) 2005-2006. Psychiatry Res. 2019;271:732-9. Epub 2019/02/23. doi: 10.1016/j.psychres.2018.11.004. PubMed PMID: 30791349.

5.Mao Y, Weng J, Xie Q, Wu L, Xuan Y, Zhang J, et al. Association between dietary inflammatory index and Stroke in the US population: evidence from NHANES 1999-2018. BMC Public Health. 2024;24(1):50. Epub 2024/01/04. doi: 10.1186/s12889-023-17556-w. PubMed PMID: 38166986; PubMed Central PMCID: PMCPMC10763382.

6.National Health and Nutrition Examination Survey Biospecimen Program.Serum, Plasma, and Urine Specimens. <https://www.cdc.gov/nchs/nhanes/biospecimens/biospecimens.htm>. Accessed 1 September 2023.

7.Shivappa N, Steck SE, Hurley TG, Hussey JR, Hébert JR. Designing and developing a literature-derived, population-based dietary inflammatory index. Public Health Nutr. 2014;17(8):1689-96. Epub 2013/08/15. doi: 10.1017/s1368980013002115. PubMed PMID: 23941862; PubMed Central PMCID: PMCPMC3925198.

8.Shivappa N, Steck SE, Hurley TG, Hussey JR, Ma Y, Ockene IS, et al. A population-based dietary inflammatory index predicts levels of C-reactive protein in the Seasonal Variation of Blood Cholesterol Study (SEASONS). Public Health Nutr. 2014;17(8):1825-33. Epub 2013/10/11. doi: 10.1017/s1368980013002565. PubMed PMID: 24107546; PubMed Central PMCID: PMCPMC3983179.
